# Cardiac structural changes after transcatheter aortic valve replacement: systematic review and meta-analysis of cardiovascular magnetic resonance studies

**DOI:** 10.1186/s12968-020-00629-9

**Published:** 2020-06-01

**Authors:** Ghazaleh Mehdipoor, Shmuel Chen, Saurav Chatterjee, Pooya Torkian, Ori Ben-Yehuda, Martin B. Leon, Gregg W. Stone, Martin R. Prince

**Affiliations:** 1 Cardiovascular Research Foundation, New York, NY USA; 2 Department of Cardiology, Columbia University College of Physicians and Surgeons, New York, NY USA; 3 Hoffman Heart Institute, Saint Francis Hospital of the University of Connecticut, Hartford, CT USA; 4 Shahid Beheshti University of Medical Sciences, Tehran, Iran; 5 The Zena and Michael A. Wiener Cardiovascular Institute, Icahn School of Medicine at Mount Sinai, New York, NY USA; 6 Department of Radiology, Weill Cornell Medical College& New York Presbyterian Hospital, 416 East 55th Street, New York, NY 10022 USA; 7 Department of Radiology, Columbia University College of Physicians and Surgeons, New York, NY USA

**Keywords:** Transcatheter aortic valve replacement (TAVR), Transcatheter aortic valve implantation (TAVI), Aortic stenosis, Magnetic resonance imaging (MRI), Cardiac structure

## Abstract

**Background:**

Transcatheter aortic valve replacement (TAVR) is increasingly used to treat patients with severe aortic stenosis (AS). Cardiovascular magnetic resonance imaging (CMR) provides reliable and reproducible estimates for assessment of cardiac structure and function after TAVR. The goal of this study was to conduct a systematic review and meta-analysis of the literature to assess left ventricular (LV) volumes, mass and function by CMR after TAVR.

**Methods:**

Using Meta-analysis of Observational Studies in Epidemiology (MOOSE) guidelines, we searched PubMed and Embase for studies reporting CMR findings before and at least 1 month after TAVR. Main factors of interest were LV end-diastolic volume index (LVEDVi), LV end-systolic volume index (LVESVi), LV mass index (LVMi), and left ventricular ejection fraction (LVEF). Standardized mean differences (SMD) were pooled by random effects meta-analytic techniques.

**Results:**

Of 453 screened publications, 10 studies (published between 2012 and 2018) were included. A total of 305 patients completed pre- and post-TAVR follow-up CMR (mean age range 78.6–85.0 years, follow-up range 6–15 months). Random effects analysis showed TAVR resulted in reduced LVEDVi (SMD: -0.25, 95% CI: − 0.43 to − 0.07, *P* = 0.006), LVESVi (SMD: -0.24, 95% CI: − 0.44 to − 0.05, *P* = 0.01), LVMi (SMD: -0.82, 95% CI: − 1.0 to − 0.63, *P* < 0.001) and increased LVEF (SMD: 22, 95% CI: 6 to 38%, *P* = 0.006). Heterogeneity across studies was low (I^2^: 0%, P_heterogeneity_ > 0.05 for all). The median reduction was 4 ml/m^2^ (IQR: 3.1 to 8.2) for LVEDVi, 5 ml/m^2^ (IQR: 3.0 to 6.0) for LVESVi, and 15.1 g/m^2^ (IQR: 11.8 to 18.3) for LVMi. The median increase for LVEF was 3.4% (IQR 1.0 to 4.6%).

**Conclusions:**

CMR demonstrates reverse LV remodeling occurrs within 6–15 months after TAVR, with reductions in LVEDVi, LVESVi and LVMi, and increased LVEF.

## Introduction

Aortic stenosis (AS) is the most common valvular heart disease in the developed world, with increased prevalence in the aging population [1]. AS is associated with cardiac remodeling due to the pressure overload including a compensatory gradual left ventricular (LV) hypertrophy, impaired LV diastolic filling, which may ultimately lead to compromised LV function in some patients [2–5]. Surgical aortic valve replacement has been the standard of care for treatment of AS for many years; however, during the past decade, transcatheter aortic valve replacement (TAVR) is increasingly used to treat patients with severe AS, especially when there are co-morbidities that increase the surgical risk [6].

Monitoring cardiac structure and function post-TAVR is usually performed by transthoracic echocardiography (TTE), which is widely available and portable. However, TTE is prone to inter-observer variability, and limited in several patient subgroups such as obese patients or patients with hyper-inflated lungs [7].

Cardiovascular magnetic resonance imaging (CMR) provides more reliable estimates for assessment of cardiac structure and function and is the non-invasive gold standard imaging modality for these purposes [8]. However, prior CMR studies on cardiac remodeling post-TAVR have been small, with variability in the results across some studies [9–11]. These limitations have led to uncertainty about the presence and magnitude of changes in ventricular structure and function after TAVR as assessed by CMR [12]. Therefore, we conducted a systematic review and meta-analysis to evaluate the changes in LV volumes, mass, and function assessed by CMR in patients undergoing TAVR.

## Methods

### Data source

We searched PubMed and Embase for original studies that reported CMR findings before and at least 1 month post-TAVR, from January 1, 2000 to August 23, 2018, using a combination of Medical Subject Heading (MeSH) terms and keywords, in accordance with Meta-analysis of Observational Studies in Epidemiology (MOOSE) guidelines (Supplementary Table 1) [13]. The MOOSE guidelines have been developed to help with standardized data design, data abstraction, meta-analysis, and reporting of observational studies. The guidelines also address some of the challenges of the observational studies such as the differences in study design, inherent biases (e.g. selection bias), and confounding factors such as varying disease severity [14].

### Study selection

All titles and abstracts of the retrieved studies were screened and where needed, the full manuscript text was reviewed for inclusion. We also searched the reference lists of included studies to identify additional potentially relevant studies. We included all cohort studies and case series that reported CMR findings before and at least 1 month after TAVR. The choice of the time interval was by authors’ consensus, so that there would be sufficient time for acute changes such as myocardial edema to resolve, and for reverse remodeling to occur [12, 15]. No language restriction nor sample size restriction were applied in selecting the articles. We excluded non-human studies, as well as ex-vivo heart assessments. Where the data from the same center were presented in multiple publications, we only included the results of the most comprehensive report from that center to avoid duplicate data.

### Data extraction and study outcomes

For each study, the data screening and abstraction was performed by 2 independent investigators (GM, and PT). All discrepancies were resolved by consensus. In addition, the investigators of primary studies were contacted for clarifications or additional data, where relevant data were missing. Main factors of interest were LV end-diastolic volume index (LVEDVi), LV end-systolic volume index (LVESVi), LV mass index (LVMi), and left ventricular ejection fraction (LVEF).

### Quality assessment

Assessing the quality of included studies, especially the appropriate reporting of the methodological details is critical for interpretation of the meta-analysis results. We assessed the methodological quality of the included full-text studies using the Newcastle-Ottawa scale as recommended by Cochrane Collaboration [16]. The Newcastle-Ottawa scale scores each study to a maximum of 9, on 3 main areas of the selection of the study groups, comparability of the groups, and the ascertainment of the outcome of interest [17].

### Statistical analysis

Standardized mean differences (SMD) from the included studies were pooled by conventional random effects meta-analytic techniques, to account for the variations across the studies. Random effects meta-analysis assumes that the estimated effects in different studies are not identical [18]. The degree of heterogeneity across studies was assessed by the I-squared (I^2^). The I^2^ < 25% was considered as low heterogeneity, and I^2^ > 75% as high heterogeneity [19]. We made a priori plans not to pool the data for variables that were reported by fewer than 3 studies. To account for possible differences in studies over time, we also performed a random effects meta-regression analysis, with year of publication and duration of follow-up as covariates, to verify the robustness of the main results. We used funnels plot and the Harbord’s regression modification of Egger’s test to assess potential publication bias for the small study effect [20]. A two-sided *P*-value of < 0.05 was considered as significant. We used STATA software, version 12.0 (StataCorp, College Station, Texas, USA) for all analyses.

## Results

**Fig. 1:**
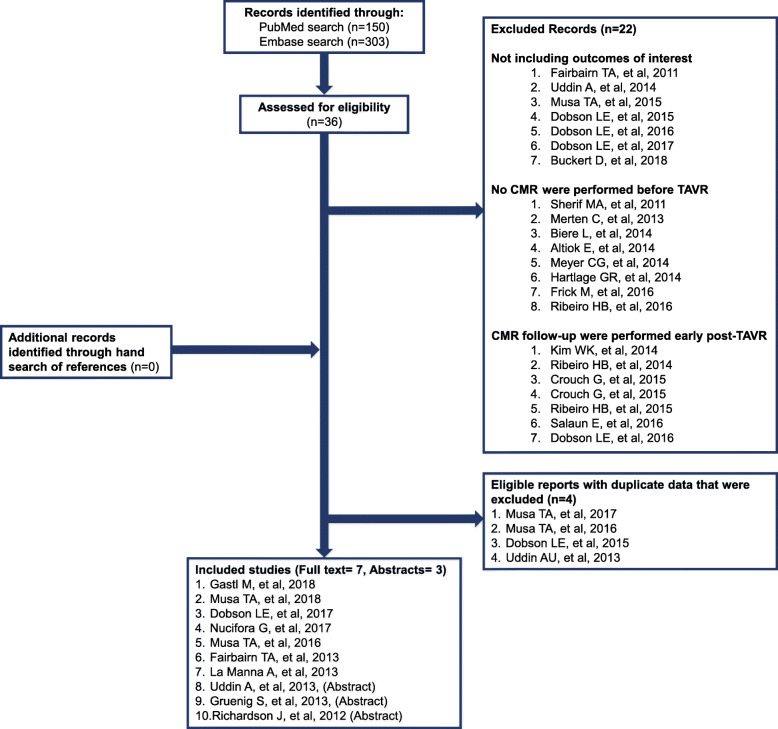
Flow diagram of included studies

A total of 453 publications were identified through our search of PubMed (150 records), and Embase (303 records). Following the screening of the titles and abstracts, 36 articles were selected for full-text review. No additional records were found through hand search of the reference lists of the retrieved articles. Finally, after excluding articles not meeting inclusion criteria or duplicate studies [21–24], 10 studies with a total of 12 study cohorts were included for meta-analysis (Fig. 1) [9–11, 25–31]. The studies were published between 2012 and 2018, and included a total of 305 patients who completed both pre- and post TAVR CMR. For all studies, follow-up CMR was performed at least 6-month post-TAVR, and all studies were performed at 1.5 T using a standard CMR protocol using the balanced steady-state free precession pulse sequence (Table 1).

**Table 1:** Summary of the study characteristics

Study, Year	Summary	Number of patients with pre- and post-TAVR CMR	Follow-up (months)	Age	Male (%)	Diabetes n (%)	Previous MI n (%)	Baseline LVEF (%)	LVEF (%) at Late Follow-up	Valve Prosthesis	Early post-TAVR CMR findings (within 7 days post-TAVR)	Excluded patients, Explanation
Gastl M, et al. 2018 [25]	43 patients with severe AS underwent TAVR.	29	6	81.9 ± 4.9	43%	12 (28)	5 (12)	58.1 ± 16.1	62.9 ± 10.4	CoreValve, Edwards Sapien	–	14 patients (poor image quality, left bundle branch block)
^a^Musa TA, et al. 2018 [10]	59 patients with severe AS underwent TAVR with either CoreValve (*n* = 27) or Lotus (*n* = 32).	19	6	79.6 ± 6.3	63%	2 (11)	3 (16)	56.2 ± 12.8	56.4 ± 8.6	CoreValve	CMR findings at median 4 days: No significant changes to LVEDVi and LVEF (*p* = 0.46 and *p* = 0.84) Significant reduction of LVMi (75.4 ± 15.0 vs. 65.8 ± 13.6 g/m^2^, *p* < 0.001)	15 patients (Death, PPM, withdrawal)
		25	6	78.6 ± 8.7	56%	5 (20)	6 (24)	51.0 ± 19.0	54.1 ± 9.3	Lotus	No significant changes to LVEDVi and LVEF (*p* = 0.55 and *p* = 0.49) No significant changes to LVMi (70.8 ± 25.0 vs 69.6 ± 16.2 g/m^2^, *p* = 0.81)	
Dobson LE, et al. 2017 [9]	88 patients with severe AS underwent TAVR. Ultimately, 24 patients with new left bundle branch block who were matched with 24 patients with a narrow post-procedure QRS were assessed.	24	6	79.6 ± 9.6	54%	2 (8.3)	2 (8.3)	56.6 ± 10.5	54.4 ± 9.3	CoreValve, Lotus	–	40 patients were excluded for various reasons, (Death, PPM, withdrawal, right bundle branch block, post-procedural myocardial infarction in 23 patients; and an additional 17 because they were not matched to a control group. CMR results of those patients were not presented)
		24	6	80.5 ± 6.2	54%	5 (20.8)	5 (20.8)	54.1 ± 11.5	58.7 ± 9			
Nucifora G, et al. 2017 [26]	59 patients with severe AS underwent either TAVR (*n* = 35) or SAVR (*n* = 24). For the current manuscript, only the TAVR cohort was considered.	17	15 ± 4	85 ± 6	63%	–	NA	68 ± 16	70 ± 14	Edwards Sapien XT	CMR findings at 4.7 ± 4 days: No significant changes to LVEF, and LVMi (*p* = 0.63 and *p* = 0.27)	18 patients (not mentioned)
^a^Musa TA, et al. 2016 [11]	167 patients with severe AS underwent TAVR (*n* = 101), or SAVR (*n* = 66). For the current manuscript, only the TAVR cohort was considered.	56	6	80.4 ± 6.6	57%	11 (20)	11 (20)	52 ± 13	53 ± 11	CoreValve, Lotus	–	45 patients (Death, PPM, withdrawal, valvuloplasty, medical therapy, claustrophobia)
Fairbairn TA, et al.2013 [27]	77 patients with severe AS underwent TAVR (*n* = 50) or SAVR (n = 27). For the current manuscript, only the TAVR cohort was considered.	25	6	80 ± 6	56%	7 (28)	5 (20)	52 ± 12	56 ± 10	CoreValve	–	25 patients (Death, PPM, withdrawal, valvuloplasty, medical therapy, claustrophobia)
La Manna A, et al. 2013 [28]	39 patients with severe AS underwent TAVR	27	6	80.7 ± 5.2	37%	4 (14.8)	7 (25.9)	61.5 ± 14.5	65.1 ± 7.2	CoreValve, Edwards-Sapien	–	12 patients (Death, PPM, withdrawal)
Uddin A, et al. 2013 (abstract) [24]	25 patients with severe AS had TAVR and underwent CMR.	25	6	80.6 ± 6.6	56%	–	–	52.1 ± 11.8	55.9 ± 9.6	CoreValve	–	–
Gruenig S, et al. 2013 (abstract) [30]	20 patients with severe AS underwent TAVR and had CMR.	20	6	83.6 ± 10.5	–	–	–	55 ± 21	63 ± 39	CoreValve, Edwards-Sapien	–	–
Richardson J, et al. 2012 (abstract) [31]	14 patients with severe AS underwent TAVR and had CMR.	14	10	–	–	–	–	54 ± 4	57 ± 5	CoreValve, Edwards-Sapien	–	–

*CMR* Cardiovascular magnetic resonance, *TAVR* Transcatheter aortic valve replacement, *SAVR* Surgical aortic valve replacement, *HTN* hypertension, *MI* Myocardial infarction, *LVEF* Left ventricular ejection fraction, *PPM* Permanent pacemaker

Please note that not all patients with available baseline characteristics in included studies had follow-up CMR available

^a^These 2 publication included 100 patients of whom 7 were repeated

### Left ventricular changes

CMR at follow-up showed a significant reduction in LVEDVi (9 cohorts; 242 patients; SMD: -0.25, 95% CI: − 0.43 to − 0.07, *P* = 0.006). No evidence of heterogeneity was observed between the included studies (I^2^: 0%, P_heterogeneity_ = 0.97) (Fig. 2a). The median study-level average reduction in LVEDVi was 4.0 ml/m^2^ (interquartile range [IQR]: 3.1 to 8.2).

**Fig. 2:**
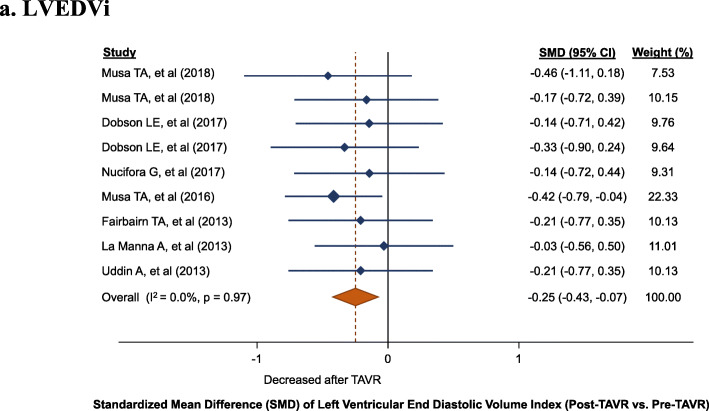
Left ventricular (LV) changes, standardized mean difference (SMD) post-TAVR vs. pre-TAVR for: **a**. LVEDVi, **b**. LVESVi, **c**. LVMi, **d**. LVEF. LVEDVi, left ventricular end-diastolic volume index; LVEF, left ventricular ejection fraction; LVESVI, left ventricular end-systolic volume index, LVMi, left ventricular mass index; TAVR, transcatheter aortic valve replacement

**Fig. 2:**
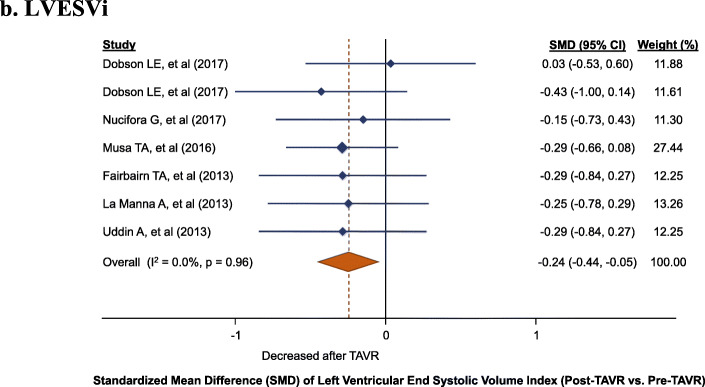
Left ventricular (LV) changes, standardized mean difference (SMD) post-TAVR vs. pre-TAVR for: **a**. LVEDVi, **b**. LVESVi, **c**. LVMi, **d**. LVEF. LVEDVi, left ventricular end-diastolic volume index; LVEF, left ventricular ejection fraction; LVESVI, left ventricular end-systolic volume index, LVMi, left ventricular mass index; TAVR, transcatheter aortic valve replacement

**Fig. 2:**
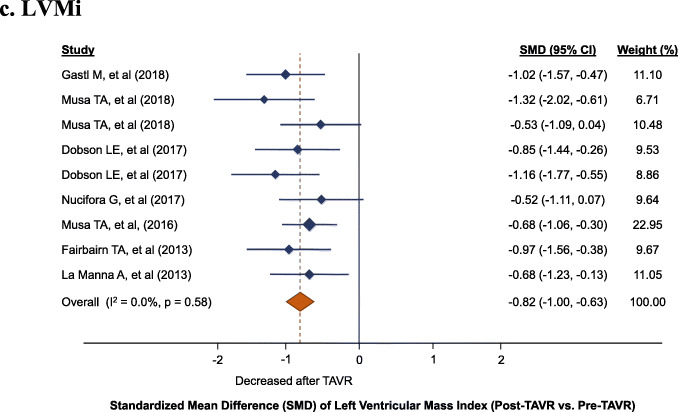
Left ventricular (LV) changes, standardized mean difference (SMD) post-TAVR vs. pre-TAVR for: **a**. LVEDVi, **b**. LVESVi, **c**. LVMi, **d**. LVEF. LVEDVi, left ventricular end-diastolic volume index; LVEF, left ventricular ejection fraction; LVESVI, left ventricular end-systolic volume index, LVMi, left ventricular mass index; TAVR, transcatheter aortic valve replacement

**Fig. 2:**
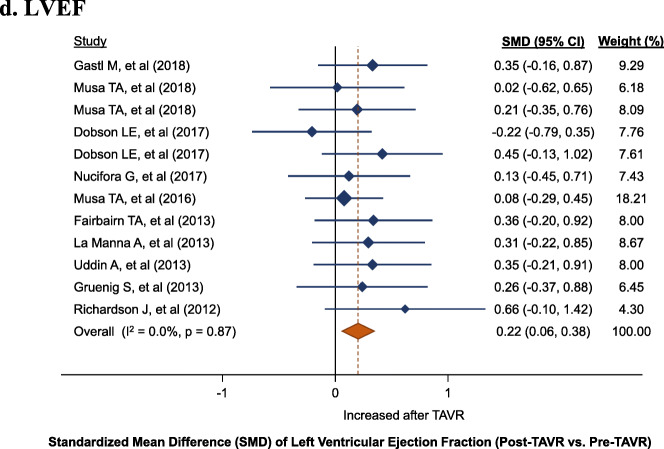
Left ventricular (LV) changes, standardized mean difference (SMD) post-TAVR vs. pre-TAVR for: **a**. LVEDVi, **b**. LVESVi, **c**. LVMi, **d**. LVEF. LVEDVi, left ventricular end-diastolic volume index; LVEF, left ventricular ejection fraction; LVESVI, left ventricular end-systolic volume index, LVMi, left ventricular mass index; TAVR, transcatheter aortic valve replacement

LVESVi was also reduced after TAVR (7 cohorts; 198 patients; SMD: -0.24, 95% CI: − 0.44 to − 0.05, *P* = 0.01) with no evidence of heterogeneity (I^2^: 0%, P_heterogeneity_ = 0.96) (Fig. 2b). The median study-level average reduction for LVESVi was 5.0 ml/m^2^ (IQR: 3.0 to 6.0).

LVMi was significantly reduced after TAVR (9 cohorts; 246 patients; SMD: -0.82, 95% CI: − 1.0 to − 0.63, *P* < 0.001). Across the included studies, no evidence of heterogeneity was observed (I^2^: 0%, P_heterogeneity_ = 0.58) (Fig. 2c). The median average reduction for LVMi was 15.1 g/m^2^ (IQR: 11.8 to 18.3).

LVEF was significantly increased after TAVR (12 cohorts; 305 patients; SMD: 22, 95% CI: 6 to 38%, *P* = 0.006). Among the included studies, there was no evidence of heterogeneity (I^2^: 0%, P_heterogeneity_ = 0.87) (Fig. 2d). Across the studies, the median average LVEF at baseline was 54.6% (IQR: 52.0 to 58.1%) and the median average increase was 3.4% (IQR 1.0 to 4.6%).

### Sensitivity analysis

In a post-hoc sensitivity analysis, we repeated the analyses for LVEDVi, LVESVi, LVMi, and LVEF after excluding 3 studies that were presented as abstracts. Although the point estimates had modest changes, the results were consistent, suggestive of significant reduction in LVEDVi, LVESVi, and LVMi, and improvement in LVEF (Table 2, Supplementary Figure 1).

**Table 2:** Left ventricular changes, degree of heterogeneity (I^2^) across the included studies after excluding 3 studies that were presented as abstracts

	SMD (95%CI)	*P*-value	I^2^	P-heterogeneity
LVEDVi	0.23 (0.03–0.44)	0.02	0.0%	0.91
LVESVi	0.25 (0.07–0.43)	0.007	0.0%	0.94
LVMi	0.80 (0.62–0.98)	< 0.001	0.0%	0.52
LVEF	18% (1–35%)	0.04	0.0%	0.82

*SMD* Standardized mean difference, *LVEDVi* Left ventricular end diastolic volume index, *LVESVi* Left ventricular end systolic volume index, *LVMi* Left ventricular mass index, *LVEF* left ventricular ejection fraction

### Risk of bias assessment

The quality of included studies was assessed using the Newcastle-Ottawa Scale. The total score varied between 6/9 and 8/9. The most frequent limitations were the lack of representation of the exposed cohort, and incomplete follow-up (Supplementary Table 2).

### Publication bias

No publication bias was apparent from visual inspection of the funnel plots for LVEDVi, LVESVi, LVEF, and LVMi (Supplementary Figure 2) and this was confirmed quantitatively with the Harbord’s regression modification of Egger’s test (*P* > 0.25 for all).

### Meta-regression

Meta-regression analysis did not show a significant association between either the duration of follow-up post-TAVR or the year of study publication and any of LVEDVi, LVESVi, LVEF and LVMi measurements (*P* > 0.25 for all) (Supplementary Table 3).

## Discussion

This systematic review and meta-analysis indicates that among patients undergoing TAVR who completed the follow-up CMR 6–15 months after the procedure, there was evidence for reverse LV remodeling. The decrease observed in LVEDVi and LVESVi likely reflects improved preload and afterload. Further, the significant reduction in LVMi toward normal values, along with the increased LVEF are consistent with gradual reverse LV remodeling after TAVR. This study adds to the existing literature by providing comprehensive estimates of association using the existing published data from prior individual studies on the topic [9–11, 25–31].

The prognostic impact of maladaptive cardiac remodeling, as well as reverse remodeling are well recognized in several cardiac conditions [32–34]. Interestingly, prior studies have indicated that irreversibility of changes or lack of improvement in LVEDVi, and LVMi post-TAVR are associated with worse outcomes [35]. Whereas an improvement in these indices is associated with better survival [35–37]. Despite the value of those studies, such investigations were based on TTE, which is subject to image acquisition limitations, as well as inter-observer and intra-observer variability [38]. To the best of our knowledge, this is the first systematic review of the literature to assess the cardiac structural changes by CMR, post-TAVR. These results are complementary to the existing TTE findings and provide additional evidence of cardiac reverse remodeling post-TAVR.

Our findings based on pooled results from CMR studies are consistent with the previous TTE findings for LVMi. TTE data from PARTNER trial have shown continuous regression of LVMi (g/m^2^) over time (from 155.6 ± 40.6 at baseline, to 148.6 ± 38.3 at 30-day follow-up, to 140.4 ± 38.1 at 6-month follow-up, to 135.7 ± 37.9 at 1-year follow-up, and to 124.7 ± 35.6 at 2-year follow-up) [37]. Further, TTE data from the PARTNER trial have shown improvement in LVEF (%) after TAVR (52.6% ± 13.4 at baseline, to 56.0% ± 11.2 at 30-day follow-up, and to 56.6% ± 10.4 at 1-year follow-up) [37].

### Limitations

Our study has several limitations. First, it is possible that some reports were not retrieved through our search strategy or were not indexed in PubMed. Second, it is clinically relevant to understand the pattern of reverse remodeling in certain patient subgroups, including those with concentric hypertrophy without LV dilation. In addition, mitral regurgitation is common in patients undergoing TAVR, and presence and severity of mitral regurgitation may also influence the remodeling [39]. However, detailed clinical and CMR data across the studies were not available to address this issue. Third, data regarding the valve type, and its potential effect on the cardiac remodeling was not available across the included studies. Fourth, the vast majority of included studies assessed the CMR characteristics 6 months post-TAVR. Additional studies are required to report post-TAVR CMR features in other time intervals. Fifth, there were a few (7/100) overlapping patients among 2 of the included studies. However, given the total number of patients in the pooled sample (*N* = 305), it is unlikely that such a modest overlap substantively impacted the results. Sixth, across the included studies, several patients were excluded from follow-up CMR post-TAVR due to permanent pacemaker implantation, death, or claustrophobia. It is possible that the patients who died or who had pacemakers have a different remodeling response to TAVR. This hypothesis is supported by subgroup analyses of prior relatively small TTE studies in patients with pacemakers, post-TAVR [40]. With the availability of CMR compatible pacemakers, as well as promising results of a recent investigation about the relative safety of CMR even in patients with traditional pacemaker devices [41], it is possible that future CMR studies further assess these subgroups.

## Conclusions

In conclusion, CMR demonstrates reverse LV remodeling within 6–15 months after TAVR, with reductions in LVEDVi, LVESVi and LVMi, and improved LVEF. Better understanding of the reverse remodeling process among patients with different comorbidities, and the prognostic utility of these findings warrant further investigation.

## Supplementary information

**Additional file 1.** Supplementary Table S1. Search strategy.

**Additional file 2.** Supplementary Table S2. Quality assessment of included studies using the Newcastle-Ottawa Scale.

**Additional file 3.** Supplementary Table S3. Meta regression for year of study publication and follow-up months.

**Additional file 4.** Supplementary Figure S1. Left ventricular changes after excluding 3 abstracts, standardized mean difference (SMD) for: a. LVEDVi, b. LVESVi, c. LVMi, d. LVEF.

**Additional file 5.** Supplementary Figure S2. Funnel plots for potential publication bias showing symmetric distribution for: a. LVEDVi, b. LVESVi, c. LVMi, d. LVEF.

## Data Availability

The datasets used and analyzed during the current study are available from the corresponding author on reasonable request.

## Abbreviations

AS: Aortic stenosis
CI: Confidence interval
CMR: Cardiovascular magnetic resonance
EDVi: End-diastolic volume index
EF: Ejection fraction
ESVi: End systolic volume index
HTN: Hypertension
LV: Left ventricle/left ventricular
LVMi: LV mass index
MeSH: Medical subject heading
MOOSE: Meta-analysis of Observational Studies in Epidemiology
SAVR: Surgical aortic valve replacement
SMD: Standardized mean differences
TAVR: Transcatheter aortic valve replacement
TTE: Transthoracic echocardiography
