# Supplementary material for: Cardiac structural changes after transcatheter aortic valve replacement: systematic review and meta-analysis of cardiovascular magnetic resonance studies
Source: J Cardiovasc Magn Reson. 2020 Jun 1;22:41. doi: 10.1186/s12968-020-00629-9 (PMC7262773; doi:10.1186/s12968-020-00629-9)
Supplement: Supplementary file 1 — Additional file 1. Supplementary Table S1. Search strategy. [file 12968_2020_629_MOESM1_ESM.docx]

| **Supplementary Table 1. Search strategy** | |
| --- | --- |
| PubMed | ("Magnetic Resonance Imaging"[MAJR] OR “Magnetic Resonance Imaging”[TIAB] OR "Magnetic Resonance Angiography"[MAJR] OR (Magnet*[TI] AND resonan*[TI])) AND ("Transcatheter Aortic Valve Replacement"[MAJR] OR “Transcatheter Aortic Valve Implantation”[TIAB] OR ((transcathet*[TI] OR trans-catheter [TI]) AND aortic*[TI]) OR TAVR*[TIAB] OR TAVI*[TIAB] OR "Transcatheter Aortic Valve Replacement/adverse effects"[MAJR]) |
| Embase | ('transcatheter aortic valve implantation':ti OR 'tavr':ti OR 'tavi':ti) AND ('magnetic resonance imaging'/exp OR 'magnetic resonance imaging' OR (magnetic AND ('resonance'/exp OR resonance) AND ('imaging'/exp OR imaging))) NOT ('cerebral':ti OR 'brain':ti) |
