# Supplementary material for: Cardiac structural changes after transcatheter aortic valve replacement: systematic review and meta-analysis of cardiovascular magnetic resonance studies
Source: J Cardiovasc Magn Reson. 2020 Jun 1;22:41. doi: 10.1186/s12968-020-00629-9 (PMC7262773; doi:10.1186/s12968-020-00629-9)
Supplement: Supplementary file 2 — Additional file 2. Supplementary Table S2. Quality assessment of included studies using the Newcastle-Ottawa Scale. [file 12968_2020_629_MOESM2_ESM.docx]

| **Supplementary Table 2. Quality assessment of included studies using the Newcastle-Ottawa Scale** | | | | | | | | | |
| --- | --- | --- | --- | --- | --- | --- | --- | --- | --- |
| Authors, Year | Selection | | | | Comparability of cohorts | Outcome | | | Total score |
|  | Representativeness of exposed cohort | Selection of non-exposed cohort | Ascertainment of exposure | Outcome not present at baseline |  | Assessment of outcome  (blindness) | Sufficient follow-up duration | Adequate follow-up |  |
| Gastl M, et al, 2018 | - | * | * | * | ** | Not mentioned | * | - | 6/9 |
| Musa TA, et al, 2018 | - | * | * | * | ** | * | * | - | 7/9 |
| Dobson LE, et al, 2017 | - | * | * | * | ** | * | * | * | 8/9 |
| Nucifora G, et al, 2017 | - | * | * | * | ** | Not mentioned | * | - | 6/9 |
| Musa TA, et al, 2016 | - | * | * | * | ** | * | * | - | 7/9 |
| Fairbairn TA, et al, 2013 | - | * | * | * | ** | * | * | * | 7/9 |
| La Manna A, et al, 2013 | - | * | * | * | ** | * | * | - | 7/9 |
| Uddin A, et al, 2013 (abstract) | - | * | * | * | ** | Not mentioned | * | * | 7/9 |
| Gruenig S, 2013 (abstract) | - | * | * | * | ** | Not mentioned | * | * | 7/9 |
| Richardson J, 2012 (abstract) | - | * | * | * | ** | Not mentioned | * | * | 7/9 |
