# Supplementary material for: Cardiac structural changes after transcatheter aortic valve replacement: systematic review and meta-analysis of cardiovascular magnetic resonance studies
Source: J Cardiovasc Magn Reson. 2020 Jun 1;22:41. doi: 10.1186/s12968-020-00629-9 (PMC7262773; doi:10.1186/s12968-020-00629-9)
Supplement: Supplementary file 3 — Additional file 3. Supplementary Table S3. Meta regression for year of study publication and follow-up months. [file 12968_2020_629_MOESM3_ESM.docx]

| **Supplementary Table 3. Meta regression for year of study publication and follow-up months** | | |
| --- | --- | --- |
| CMR findings | Follow-up (months)  P-value | Year of study publication  P-value |
| Left ventricular end diastolic volume index (LVEDVi) | 0.67 | 0.53 |
| Left ventricular end systolic volume index (LVESVi) | 0.84 | 0.85 |
| Left ventricular ejection fraction (LVEF) | 0.85 | 0.29 |
| Left ventricular mass index (LVMi) | 0.36 | 0.65 |
