# Supplementary material for: Cardiac structural changes after transcatheter aortic valve replacement: systematic review and meta-analysis of cardiovascular magnetic resonance studies
Source: J Cardiovasc Magn Reson. 2020 Jun 1;22:41. doi: 10.1186/s12968-020-00629-9 (PMC7262773; doi:10.1186/s12968-020-00629-9)
Supplement: Supplementary file 4 — Additional file 4. Supplementary Figure S1. Left ventricular changes after excluding 3 abstracts, standardized mean difference (SMD) for: a. LVEDVi, b. LVESVi, c. LVMi, d. LVEF. [file 12968_2020_629_MOESM4_ESM.docx]

**Supplementary Figure 1. Left ventricular changes after excluding 3 abstracts, standardized mean difference (SMD) for: a. LVEDVi, b. LVESVi, c. LVMi, d. LVEF.**

**Supplementary Figure 1. a. LVEDVi**

**
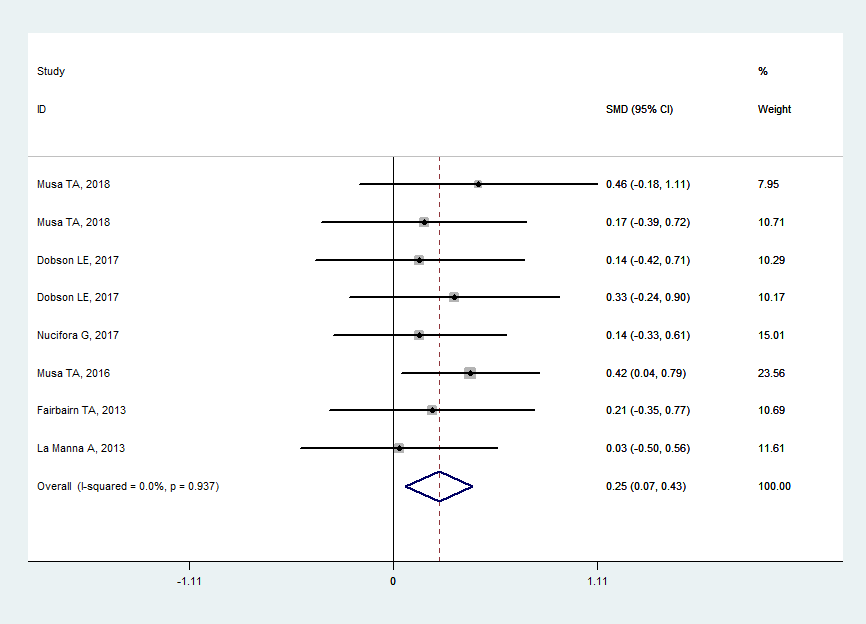
**

**Supplementary Figure 1. b. LVESVi**

**
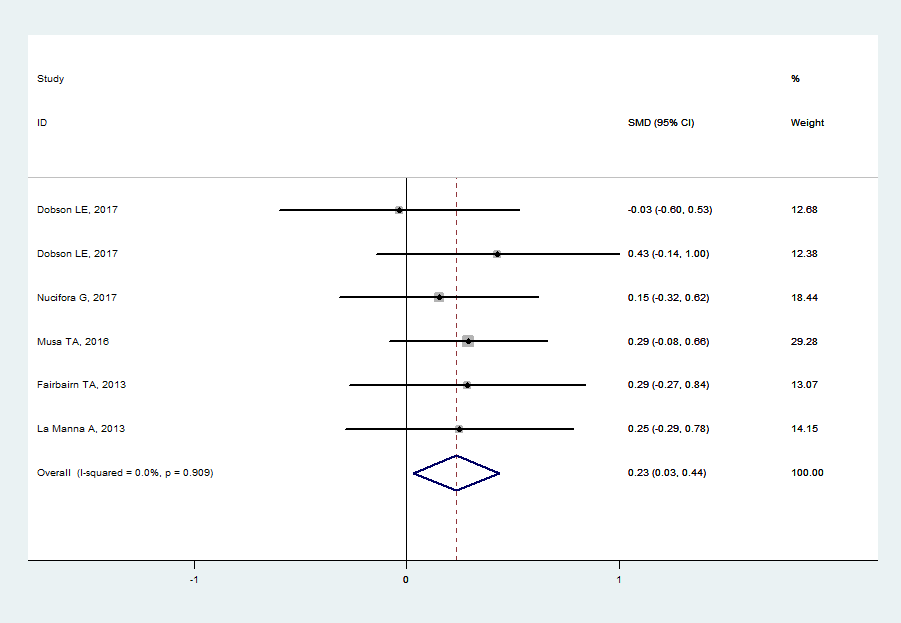
**

**Supplementary Figure 1. c. LVMi**

**
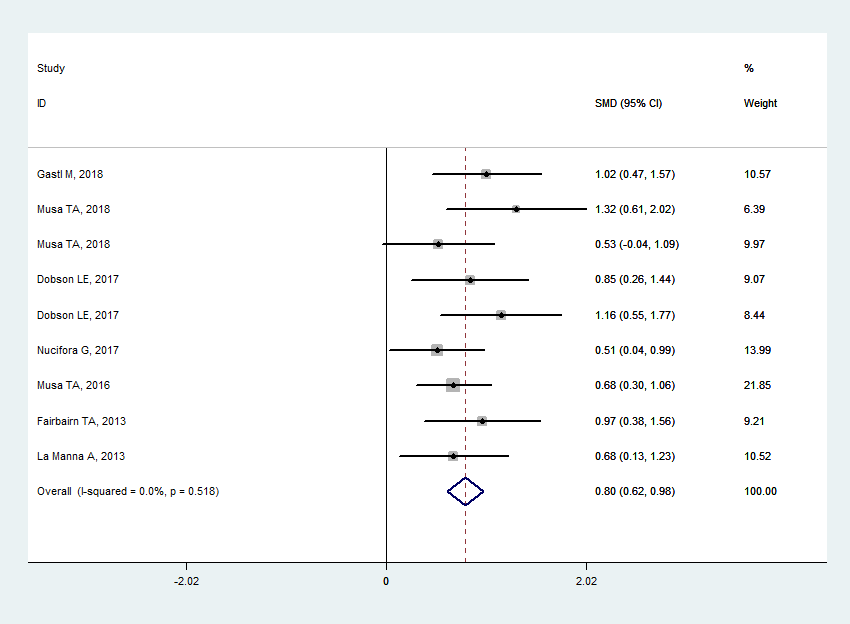
**

**Supplementary Figure 1. d. LVEF**

**
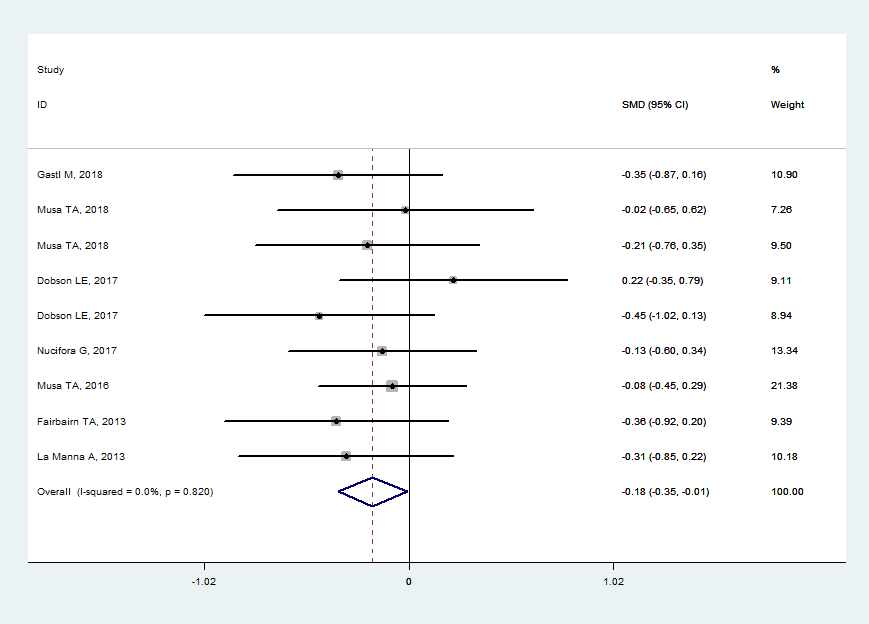
**
