# Supplementary material for: Cardiac structural changes after transcatheter aortic valve replacement: systematic review and meta-analysis of cardiovascular magnetic resonance studies
Source: J Cardiovasc Magn Reson. 2020 Jun 1;22:41. doi: 10.1186/s12968-020-00629-9 (PMC7262773; doi:10.1186/s12968-020-00629-9)
Supplement: Supplementary file 5 — Additional file 5. Supplementary Figure S2. Funnel plots for potential publication bias showing symmetric distribution for: a. LVEDVi, b. LVESVi, c. LVMi, d. LVEF. [file 12968_2020_629_MOESM5_ESM.docx]

**Supplementary Figure 2. Funnel plots for potential publication bias showing symmetric distribution**

**Supplementary Figure 2. a. LVEDVi**


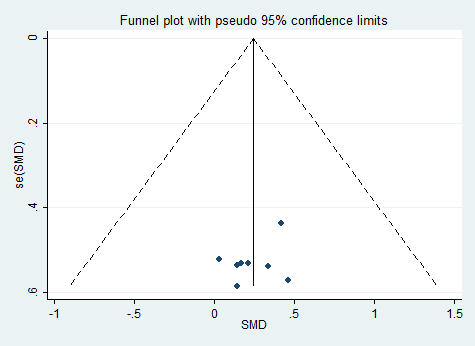


**Supplementary Figure 2. b. LVESVi**


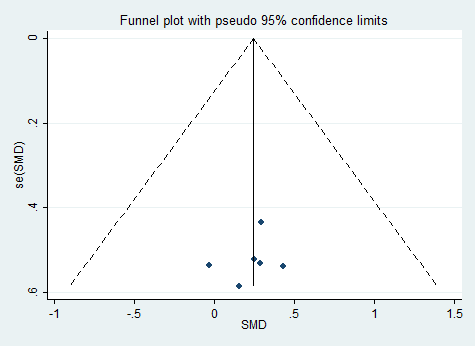


**Supplementary Figure 2. c. LVMi**

**Supplementary Figure 2. d. LVEF**
